# Long-term visual outcome in idiopathic intracranial hypertension

**DOI:** 10.4103/0972-2327.78044

**Published:** 2011

**Authors:** N. N. Baheti, M. Nair, S. V. Thomas

**Affiliations:** Department of Neurology, Sree Chitra Tirunal Institute for Medical Sciences and Technology, Trivandrum, Kerala, India

**Keywords:** Fulminant IIH, idiopathic intracranial hypertension, pseudotumor cerebri

## Abstract

**Objective:**

To characterize the course, outcome, and risk of relapse or late worsening in a clearly defined cohort of idiopathic intracranial hypertension (IIH) over a long period of follow-up.

**Materials and Methods:**

Retrospective chart review of patients with definite IIH was evaluated at the Sree Chitra Tirunal Institute for Medical Sciences and Technology between 1998 and 2006. Patients’ demographic data, clinical, neuro-ophthalmic examinations, and treatment details were abstracted. Patients were further categorized into three groups based on whether they improved, worsened, or relapsed on follow-up. Final visual outcome of each patient was defined according to grading of the worse eye at the last visit. Statistical analysis included t test to compare group means and chi-square test to compare proportions.

**Results:**

Of the 43 women included, visual impairment was observed in 80 eyes (93%) at presentation and it was moderate to severe in 14%. The mean CSF opening pressure at presentation did not differ significantly in those with visual impairment compared to those with normal vision. Those having early severe visual impairment had significantly higher (*P* = 0.015) likelihood of severe visual impairment on last follow-up. Of the total, 34 patients (79%) improved, 4 (9.3%) relapsed on follow-up after period of stability, and 5 (11.6%) worsened over 56 months follow-up (range, 26-132 months). The groups were comparable, except those who improved were younger (*P*<0.05). At last examination, 9% had significant vision loss.

**Conclusion:**

IIH patients can have delayed worsening or relapses and about tenth of patients can have permanent visual loss early or late in the course of the disease. All patients with IIH need to be kept under long-term follow-up, with regular monitoring of visual functions.

## Introduction

Idiopathic intracranial hypertension (IIH) had been viewed from different perspectives as denoted by the several eponyms it carries. The term pseudotumor cerebri had been used in view of the raised intracranial pressure and papilledema in the absence of a brain tumor.[1] The term benign intracranial hypertension had been used for several decades in consideration of the relatively benign course and likelihood of recovery without major sequelae.[1] IIH is currently the favored term for the primary (idiopathic) disorder, where no specific etiology had been readily identified.

IIH is increased intracranial pressure (ICP) with normal cerebrospinal fluid (CSF) characteristics in the absence of an intracranial mass, hydrocephalus, or other identifiable cause.[1] The incidence is approximately 0.9/100 000/year rising to 13/100 000/year in women between 20 and 44 years of age, who are 10% above ideal body weight. IIH is also seen in men and children, but less frequently. Prevalence rates are higher, reflecting the chronic nature of the condition in many cases.[2,3] IIH is associated with many conditions, but most of these associations have not been scientifically proven.[4] Its pathophysiology and treatment remain controversial. The principal consequence of IIH is visual loss, which can be irreversible if not treated promptly. IIH can have a variable course ranging from a short benign self-limiting syndrome to more aggressive syndromes that proceed to blindness in a short period of time.[5] Nevertheless, much less is known about the long-term outcome with reference to the rate of recurrence[6] or visual function.[7,8] The purpose of this study is to characterize the presenting clinical features and assess long-term visual outcome in patients with IIH.

## Materials and Methods

This study was carried out in the Neurology department of Sree Chitra Tirunal Institute for Medical Sciences and Technology, which is a tertiary referral center in South India. We identified all cases of IIH treated as inpatients in this department between 1998 and 2006 by systematically screening all the medical records. Only cases with definite IIH according to the modified Dandy criteria[1] were selected. These criteria included (1) symptoms and signs of generalized intracranial hypertension such as headache, papilledema, sixth nerve palsies; (2) documented elevated ICP; (3) normal CSF composition; and (4) no evidence of hydrocephalus, mass, or structural or vascular lesion on brain magnetic resonance (MR) imaging; specifically, no evidence of cerebral venous thrombosis. The exclusion criteria included (1) other ocular disease causing visual loss; (2) systemic conditions or medications that may be associated with intracranial hypertension; and (3) alternative plausible mechanisms of visual loss other than IIH.

Patients’ demographic characteristics and clinical details were abstracted from the medical records on to standard proforma. Presenting features (headache, pulsatile intracranial noises, diplopia and transient visual obscuration); duration of symptoms, CSF opening pressure, serial visual acuity and visual fields, treatment, and visual outcome were also recorded. MR imaging and postcontrast MR venography of brain were performed in all patients.

All patients underwent neuro-ophthalmic examinations including ophthalmoscopic examination, best-corrected visual acuity, and formal visual field testing with automated static perimetry (Humphrey), kinetic perimetry (Goldmann), or a combination of the two. The sensitivity for detection of visual field defects is similar using quantitative perimetry with static or kinetic methods.[8,9] Automated perimetry was not available for all patients, hence not taken for final analysis.

Visual impairment was determined by the simplified grading system defined by Wall and George which takes in to account visual acuity and visual field (field constriction defects involving visual fixation and defects involving the fixation). This system grades visual impairment from grade 0 (normal) to grade 5 (blinding visual loss).[9] Blindness is defined as best visual acuity less than 20/200 (6/60) or visual field less than 20° to the largest Goldmann test object (V4 = 64 mm^2^).

Patients were further categorized into three groups based on whether they improved, worsened, or relapsed on follow-up. Worsening was defined as a decrease in visual function (i.e., decrease in one grade) after an initial period of stability. Recurrence was reappearance of symptoms and signs of IIH after resolution and being off medication for IIH for at least 6 months. Final visual outcome of each patient was defined according to grading of the worse eye at the last visit. Visual functions were described for each eye separately.

All cases were initially treated with standard medical treatments for IIH including monotherapy (e.g., acetazolamide) or combination therapy (e.g., acetazolamide, furosemide, and steroids). Patients with progressive visual loss who did not respond to maximum tolerated medical treatment underwent CSF shunting procedure.

### Statistical analysis

The data were transferred to a spreadsheet and analyzed with the help of SPSS for windows software 11. We used t test to compare group means and chi-square test to compare proportions. The significance level was set to 0.05.

## Results

There were 43 patients (all females) with mean age 25.6 ± 9.2 years (range, 12 – 48 years) who satisfied the selection criteria for IIH during the study period. The mean duration of symptoms before diagnosis was 39.8 ± 61.1 days (range, 10 – 270 days). Systemic hypertension was present in three patients. The important symptoms at presentation in the order of frequency were headache (93%), scintillations (58.1%) transient visual obscurations (46.5%), persistent visual impairment (41.9%), pulsatile intracranial noises (41.9%), diplopia (32.6%), and retro-orbital pain (18.6%). The interval between the first symptom and worst visual impairment ranged from 1 to 210 days (mean, 27.1 ± 44.8 days; median, 15 days). The mean CSF opening pressure during the initial examination was 314 ± 65 mm H_2_O (range, 180 – 560 mm H_2_O). The mean duration of follow-up was 56 months (median, 50 months; range, 26 – 132 months).

### Visual examination

**Figure 1:**
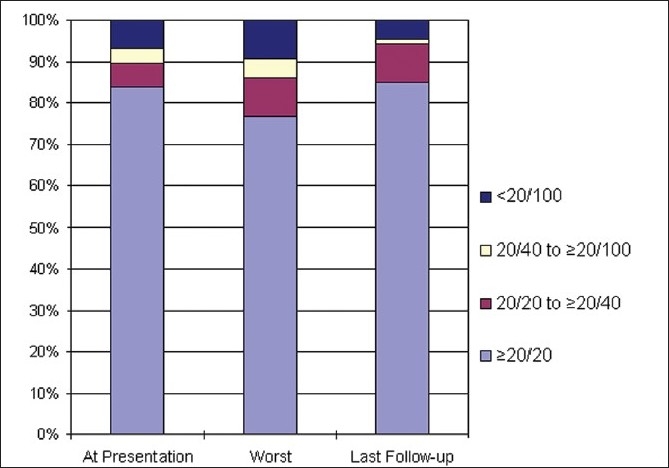
Visual acuity at different stages of follow-up

Eight-six eyes (43 patients) were evaluated in this study. Visual acuity was normal in 72 eyes (83.7%). At presentation, visual acuity was impaired in 14 eyes (16.2%) and it was less than 20/200 in four eyes (5.9%). At last follow-up, 13 eyes (15%) had visual acuity less than 20/20. Totally, 66 eyes (76%) had a visual acuity of 20/20 throughout the follow-up period. The distribution of cases according to severity of visual acuity at different time frames is as shown in Figure 1. At presentation, visual field defects were observed in 80 eyes (93%). The common defects were generalized constricted fields, enlargement of blind spot, nasal and arcuate defects. Visual impairment (Wall and George grade 1 – 5) was observed in 80 eyes (93%) at presentation. The mean CSF opening pressure at presentation for those with visual impairment (311 ± 60 mm H_2_O) did not differ significantly from that of those with normal vision (347 ± 66 mm H_2_O).

**Figure 2:**
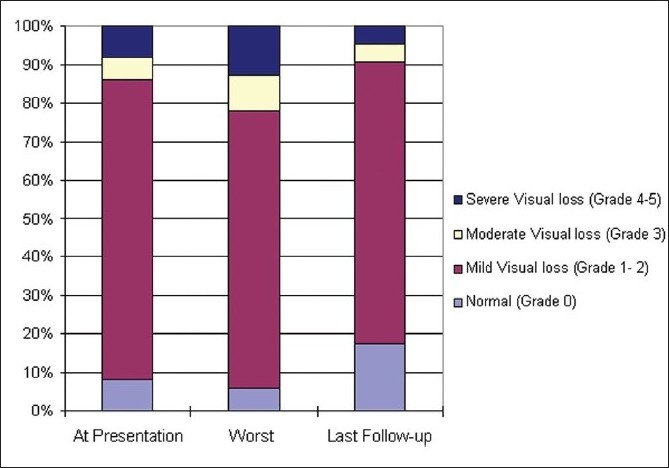
Visual impairment at different stages of follow-up

At presentation, 12 eyes (14%) had moderate to severe visual impairment (Wall and George grade 3 – 5) and 19 eyes (22%) had moderate to severe visual impairment at some or other time during the follow-up, but most of these changes were reversible, only eight eyes (9%) had such significant vision loss at last examination [Figure 2]. There were 14 patients (32.5%) with symptoms for less than 15 days and two of them had severe visual impairment (Wall and George Grade 4 or more) at presentation. A subgroup analysis of them showed that those who had early severe visual impairment (n = 2) had significantly (*P* = 0.015) higher likelihood of severe visual impairment on last follow-up.

Six patients (14%) had blinding visual impairment (Wall and George grade 5) at presentation. Three of these were subjected to shunting procedure, rest were managed pharmacologically. All except one (pharmacologically managed) had some degree of improvement at the last follow-up. The mean CSF opening pressure of those undergoing surgery (n = 3) was 357 ± 72 mm H_2_O (range, 280 – 450 mm H_2_O). Mean follow-up for this group with fulminant hypertension was 61 months (median, 61 months; range, 30 – 96 months). The characteristics at presentation compared to those with mild or no visual impairment were not statistically significant.

The patients were categorized into three groups according to their course of the disease. Of the total, 34 patients (79%) improved, four (9.3%) relapsed on follow-up after a period of stability, and five (11.6%) worsened over 168 patient-year follow-up. All the patients in relapsed and worsened group had bilateral visual deficits at presentation. The time to recurrence or delayed worsening was evenly distributed over the course of the follow-up. Of the four relapsed, three relapsed late into the course of illness; two after a symptom-free period of 34 months and one after 50 months. Of the eight eyes (four cases), majority improved at last follow-up; severe visual loss was persistent in only one eye. Of the five patients worsening on follow-up, two worsened late after 24 months. Of the 10 eyes, severe visual loss was persistent in four eyes and rest had significant visual improvement. For statistical comparison, those who relapsed or worsened were combined together. The baseline characteristics of the two groups were as shown in Table 1. Both the groups were comparable, except those who improved were younger (*P*<0.05).

**Table 1:** Comparison of the clinical characteristics of patients with IIH who had improved or worsened on follow-up

Baseline characteristics	Outcome on follow-up	
	Improved N (%)	Relapsed/ Worsened N (%)
Patients, n (% of total)	34 (79)	9 (21)
Mean age ± SD years	24 ± 8.2	31.8 ± 10.1#
CSF OP (mm of water)	308	337
Headache	32 (94.1)	8 (88.9)
Scintillations	14 (41.4)	4 (44.4)
PIN	13 (38.2)	5 (55.6)
TVO	15 (44.1)	5 (55.6)
PVI	13 (38.2)	4 (44.4)
Retro-orbital pain	6 (17.6)	2 (22.2)
Mean follow-up ± SD mo	53 ± 28	63 ± 24

CSF - Cerebrospinal fluid, OP- Opening pressure, PIN- Pulsatile intracranial noises, TVO- Transient visual obscurations, PVI- Persistent visual impairment, SD - Standard deviation

# *P*<0.05

## Discussion

IIH is no longer considered benign and is an important cause for visual impairment. Previous studies have suggested that up to 96% of patients would have visual abnormalities at some time during the course of IIH.[9] The natural history of IIH is largely unknown, although several researchers have highlighted the potential risk of permanent visual impairment in a small, but significant, proportion of patients with IIH.[5,9] An early hospital-based study that followed up 57 patients for 5 to 41 years found that 24% of them developed blindness or severe visual impairment.[10] Another study reported severe visual loss in 9% cases over 3 years.[5] In contrast, a community-based epidemiological study reported a lower rate of severe visual loss of 6%.[2] In the current study, with median follow-up period of 4.1 years, the long-term visual outcome was near normal for vast majority of patients. It was impaired in 9% patients only. These differences could be due to differences in study methodology, referral bias, and variable follow-up periods.

The tempo of visual impairment in IIH is highly variable. In majority, it typically evolves slowly with deficits arising over several weeks to months in significant proportion; IIH can have an acute course when vision is threatened very early. In the current series, 14 patients (32.5%) presented with visual symptoms of less than 2 weeks duration, and two of them had severe visual impairment (Wall and George Grade 4 or more). Early and severe visual loss (Fulminant IIH) at presentation had a higher likelihood of having persistent vision loss, which is similar to the previous literature.[11]

In the current series, surgical intervention was required in half of those who presented with blinding visual impairment (Wall and George grade 5), which resulted in good recovery and had no correlation with CSF opening pressure. Aggressive management strategy with early surgical intervention in nonresponders can preserve or improve vision in the majority.

The morbidity caused by IIH is similar to optic neuritis. The reported outcome in optic neuritis is 13%, having visual acuity of <20/25 at 5 years.[12] We noted that 15% had visual acuity <20/20 at 4.6 years. Compared with optic neuritis, IIH is poorly understood, often missed, therefore contributing to the morbidity.

There is a four-fold variation in the reported risk of recurrence of IIH ranging from 8 to 38%.[6,8,10,13] There are very few studies from India on the long-term outcome of IIH. In the current series, 11.6% had delayed worsening of visual functions and 9.3% had recurrence after initial recovery. Furthermore, all the cases in relapsed and worsened group had bilateral visual deficits at presentation. Another recent very long-term follow-up study from USA that included patients with more than ten-year follow-up had a delayed worsening in 30% and recurrence rate of 15%.[8] The higher rates in the above study could be due to self-selection, with only symptomatic or severely affected or refractory patients continuing to follow-up or referral bias, with only severe and atypical cases being referred to tertiary care centre. Also, IIH is etiologically related to obesity, nutritional preferences, and consumption of certain medicines and drugs. Hence, it is possible that IIH may have different natural history and long-term outcome in different geographic locations. Registries for IIH are likely to characterize better the natural history and risk of relapse or delayed worsening in this group of patients.

In this series, there was an association between older age and protracted course or relapse of IIH. We did not find any association between CSF opening pressure and clinical course or visual outcome, which is similar to previous literature.[5,6] One of the important factors linked to delayed relapses is recent weight gain, but as weight was not noted for all our patients, we could not analyze its influence.[14]

The temporal pattern of visual impairment in IIH with a tendency for early and rapid visual loss in some or delayed and slowly progressive visual impairment in others suggests that more than one mechanism may be involved. This is favored by the findings of a previous study[10] that many patients with IIH undergoing lumbar puncture years after onset of the disease still have ICP. It is important to bear in mind that even after several months to years of relative quiescence, patients with IIH can have delayed worsening of visual function or relapse. In this study, majority of the relapses occurred late after a period of 2 to 3 years. All IIH patients including those in remission should be informed about this and encouraged for regular follow-up and ophthalmological evaluation.

The major limitation of this study is its retrospective nature and the restriction of sampling to a tertiary care setting which might have lead to referral bias. In conclusion, about a tenth of patients with IIH can have permanent visual loss that can occur early or late in the course of the disease. Those with bilateral visual deficits at presentation are more likely to relapse or worsen on follow-up. All patients with IIH need to be kept under long-term follow-up, with regular monitoring of visual functions in order to detect any visual deterioration and timely intervention.
